# Impact of Obstructive Sleep Apnea on the Levels of Placental Growth Factor (PlGF) and Their Value for Predicting Short-Term Adverse Outcomes in Patients with Acute Coronary Syndrome

**DOI:** 10.1371/journal.pone.0147686

**Published:** 2016-03-01

**Authors:** Antonia Barcelo, Josep Miquel Bauça, Aina Yañez, Laura Fueyo, Cristina Gomez, Monica de la Peña, Javier Pierola, Alberto Rodriguez, Manuel Sanchez-de-la-Torre, Jorge Abad, Olga Mediano, Jose Amilibia, Maria Jose Masdeu, Joaquin Teran, Josep Maria Montserrat, Mercè Mayos, Alicia Sanchez-de-la-Torre, Ferran Barbé, Spanish Sleep Group

**Affiliations:** 1 Hospital Universitari Son Espases, Palma, Illes Balears, Spain; 2 Hospital Universitari Arnau de Vilanova and Santa Maria, IRBLleida, Lleida, Catalonia, Spain; 3 Hospital Universitari Germans Trias i Pujol, Badalona, Catalonia, Spain; 4 Hospital Universitario de Guadalajara, Guadalajara, Castilla-La Mancha, Spain; 5 Hospital Universitario Cruces, Bilbao, Basque Country, Spain; 6 Hospital Parc Taulí, Sabadell, Catalonia, Spain; 7 Hospital General Yagüe, Burgos, Castilla-León, Spain; 8 Hospital Clínic, Barcelona, Catalonia, Spain; 9 Hospital de la Santa Creu i Sant Pau, Barcelona, Catalonia, Spain; University of Rome Tor Vergata, ITALY

## Abstract

**Background:**

Placental growth factor (PlGF) induces angiogenesis and promotes tissue repair, and plasma PlGF levels change markedly during acute myocardial infarction (AMI). Currently, the impact of obstructive sleep apnea (OSA) in patients with AMI is a subject of debate. Our objective was to evaluate the relationships between PlGF levels and both the severity of acute coronary syndrome (ACS) and short-term outcomes after ACS in patients with and without OSA.

**Methods:**

A total of 538 consecutive patients (312 OSA patients and 226 controls) admitted for ACS were included in this study. All patients underwent polygraphy in the first 72 hours after hospital admission. The severity of disease and short-term prognoses were evaluated during the hospitalization period. Plasma PlGF levels were measured using an electrochemiluminescence immunoassay.

**Results:**

Patients with OSA were significantly older and more frequently hypertensive and had higher BMIs than those without OSA. After adjusting for age, smoking status, BMI and hypertension, PlGF levels were significantly elevated in patients with OSA compared with patients without OSA (19.9 pg/mL, interquartile range: 16.6–24.5 pg/mL; 18.5 pg/mL, interquartile range: 14.7–22.7 pg/mL; p<0.001), and a higher apnea-hypopnea index (AHI) was associated with higher PlGF concentrations (p<0.003). Patients with higher levels of PlGF had also an increased odds ratio for the presence of 3 or more diseased vessels and for a Killip score>1, even after adjustment.

**Conclusions:**

The results of this study show that in patients with ACS, elevated plasma levels of PlGF are associated with the presence of OSA and with adverse outcomes during short-term follow-up.

**Trial Registration:**

ClinicalTrials.gov NCT01335087

## Introduction

Recent data suggest that obstructive sleep apnea (OSA) is underdiagnosed in patients after acute myocardial infarction (AMI) [1]. Intermittent episodes of hypoxia and arousals cause an increase in sympathetic activity, oxidative stress, hypercoagulability and cardiac hyperexcitability that could aggravate the severity of AMI and worsen the short-term prognosis of OSA patients [2–4]. Nevertheless, a cardioprotective role of OSA in the context of AMI, via ischemic preconditioning, has also been postulated [5]. Such protection would require the activation of adaptive mechanisms, such as increased recruitment of proliferative and angiogenic endothelial progenitor cells [6].

With the emergence of novel biomarkers, it may be feasible to characterize different aspects of the pathophysiology of acute coronary syndrome (ACS) [7;8]. Placental growth factor (PlGF), a member of the vascular endothelial growth factor family (VEGF), is expressed in cells of the cardiovascular system and plays a predominant role in pathological angiogenesis without affecting quiescent vessels in healthy organs [9;10]. PlGF expression increases in the damaged human heart, and PlGF levels in blood increase after AMI [11]. Elevated PlGF levels have emerged as an important, independent marker of short-term adverse outcomes in patients with ACS [12]. In contrast, PlGF plasma levels in the acute phase after myocardial infarction (MI) have been found to be positively correlated with the degree of improvement in left ventricular function that occurs during the chronic phase of MI; this finding suggests that PlGF may be involved in repairing injured myocardial tissue [13]. Cardiac PlGF expression is induced by hypoxia, and it has been suggested that PlGF is a stress-response factor that suppresses pathological remodeling in the heart by inducing angiogenesis, cardiomyocyte growth and peripheral mobilization of mononuclear cells and bone marrow-derived stem cells towards ischemic myocardial tissue [11]. Recent evidence demonstrates that PlGF is a crucial mediator of adaptive cardiac remodeling after myocardial infarction, and it has been suggested that the effects of PlGF could form the basis of a potential therapeutic strategy in the future [14].

The purpose of this study was to assess the impact of OSA on circulating PlGF levels in patients with ACS and to determine whether PlGF levels have short-term prognostic significance in patients with OSA compared with patients without OSA.

## Materials and Methods

### Patients

The Ethics Committee of each participating center approved the study: the Comitè Ètic d’Investigació (Hospital Universitari Son Espases, Palma), the Comité Ético de Investigación Clínica de Euskadi (Hospital de Cruces, Bilbao), the Comité Ético de Investigación Clínica (Hospital Arnau de Vilanova i Santa Maria, Lleida), the Comitè Ètic d’Investigació Clínica (Hospital Germans Trias i Pujol, Barcelona), the Comité Ético de Investigación Clínica (Hospital General Universitario de Guadalajara, Guadalajara), the Comitè Ètic d’Investigació Clínica (Hospital Parc Taulí, Sabadell), the Comité Ético de Investigación Clínica de Burgos y Soria (Hospital General Yagüe, Burgos), and the Comitè Ètic d’Investigació Clínica (Hospital Clínic, Barcelona). All patients provided written, informed consent.

This is an ancillary study of the ISAACC Study (NCT01335087), a multicenter, open-label, parallel, prospective, randomized, controlled trial [15]. The ISAACC study is evaluating the effects of CPAP treatment on the incidence of new cardiovascular events in patients with an episode of ACS and OSA. The ISAACC study includes non-sleepy patients because it is unethical to fail to treat OSA patients with excessive daytime sleepiness. The first patient was included in April 2011, so in spite of newer guidelines regarding the sleep apnea definitions [16], the recruitment of patients was consistent as originally established for ISAACC, and published elsewhere [15;17]. For the present study, we included 538 patients (men and women age ≥18 years old) who were admitted for ACS to coronary care units or cardiology hospitalization wards from fourteen teaching hospitals in Spain. A polygraphic study was performed during the first 48–72 h after admission. The case (n = 312) or control (n = 226) status of each ACS patient was defined using an apnea-hypopnea index (AHI) threshold of 15 or greater. ACS was defined as the acute presentation of coronary disease, with or without ST-elevation, unstable angina, or MI type 1 [17]. The exclusion criteria for this study were the following: previous CPAP treatment; psychophysical inability to complete questionnaires; the presence of any previously diagnosed sleep disorder; >50% of apneas consisting of central apneas or the presence of Cheyne-Stokes respiration; the presence of daytime sleepiness (Epworth Sleep Scale [ESS] score >10); the presence of chronic diseases, including neoplasms, renal insufficiency (GFR<15 mL/min/1.73 m^2^), severe COPD (FEV_1_<50%), chronic depression, and other limiting chronic diseases; a medical history that could interfere with the objectives of the study; and any processes, whether cardiovascular or otherwise, that reduced life expectancy to <1 year; and patients in cardiogenic shock.

### Procedures

The diagnosis of OSA was based on the results of a sleep test, in accordance with the guidelines of the Spanish national consensus on apnea-hypopnea syndrome [18]. All participating centers used the same model of polygraph (Embletta; ResMed, Australia) for the diagnosis of OSA. Oronasal flow, thoracoabdominal movements, ECG, and pulse oximetry were recorded. Apnea is defined as an absence of airflow lasting ≥10 seconds. Hypopnea is defined as a reduction in airflow lasting ≥10 seconds and is associated with oxygen desaturation. Oxygen desaturation (ODI) is considered as a decrease in SaO2 >4%. The apnea-hypopnea index is defined as the number of apneas and hypopneas per hour of sleep. The extent of self-reported sleepiness/drowsiness was analyzed using the Spanish version of the ESS test [19]. Echocardiographic evaluations and Killip classification were performed routinely at hospital admission. During hospitalization, we evaluated the severity of ACS and each patient’s short-term prognosis, in terms of the ejection fraction, the Killip score, the number of affected vessels, the average and peak troponin levels, and complications related to the cardiovascular event itself (heart failure, reinfarction, mechanic complications such myocardial rupture, arrhythmia and stroke) and mortality. Other complications related to the ICU admission were excluded, such as infections or venous thrombosis following immobilization, since they were not due to the cardiovascular event for which the patient was admitted.

### Blood sampling and analysis

Peripheral blood samples were collected from all subjects after the polygraphic study at the time of randomization. Plasma and serum samples were stored at -80°C until analysis. Cardiac marker levels were measured by investigators who were blinded to the patients’ histories. Blood chemistry data were measured using commercially available assays. PlGF levels were measured in plasma using an enzyme-based electrochemiluminescence assay (Roche Diagnostics, Germany). The lower limit of detection was 3 pg/mL, and the upper limit of detection was 10.000 pg/mL. The intra-assay coefficient of variation was 1.1%, and the inter-assay coefficient of variation 2.6%.

### Data analysis

Data on the patients included in this study were incorporated into a password-protected database. The mean values (and standard deviations) or frequencies (and percentages) were computed to evaluate the differences between the control and OSA patients, and the significance of differences was assessed using Mann-Whitney tests (or t tests, if data were normally distributed) or chi-squared tests, respectively. OSA patients were divided into 3 groups according to AHI, and the Kruskal-Wallis test was used for comparisons among groups. Spearman´s rank correlations were calculated to assess correlations. The associations among PlGF levels, OSA, and variables related to the severity of ACS were assessed using the Mann-Whitney or chi-squared tests and linear or logistic regression models, as appropriate. Additionally, the models were adjusted for smoking status (current or former smoker vs non-smoker), body mass index, age, gender (male vs female), hypertension, diabetes and dyslipidemia.

Odds ratios, their corresponding 95% confidence intervals, and p-values were calculated using logistic regression models; adjusted p-values were also computed. The threshold for statistical significance was set at p<0.05. SPSS v19 software (IBM Corp., Armonk, NY) was used for all analyses.

## Results

The clinical and sleep-related characteristics of the patients are given in Table 1.

**Table 1:** Anthropometric, clinical and biochemical variables for patients with obstructive sleep apnea (OSA) and controls.

	Controls(n = 226)	OSA(n = 312)	p-value
Age, years	56.5±11.5	61.2±10.3	<0.001
Male, %	191 (84.5%)	250 (80.1%)	0.192
AHI, h^-1^	6.3±4.1	35.7±1 7.4	<0.001
ODI, h^-1^	9.3±17.9	24.9±16.9	<0.001
Mean SaO2, %	93.6±2.2	92.7±2.2	<0.001
Min SaO2, %	86.7±5.2	82.1±7.2	<0.001
Time with SaO2 <90%, %	4.2±11.5	11.2±18.0	<0.001
BMI, kg•m^-2^	26.1±6.1	28.3±6.2	<0.001
Glucose, mg/dL	119±53	128±59	0.078
HDL cholesterol, mg/dL	45±30	40±18	0.058
LDL cholesterol, mg/dL	116±41	110±33	0.103
Triglyceride, mg/dL	154±117	141±79	0.161
Hypertension	87 (38.5%)	178 (57.1%)	<0.001
Diabetes mellitus	50 (22.1%)	83 (26.6%)	0.243
Dyslipidemia	108 (47.8%)	134 (42.9%)	0.265
Smoker			0.163
No	57 (25.2%)	103 (32.8%)	
Yes	107 (47.3%)	134 (42.7%)	
Former smoker	62 (27.4%)	77 (24.5%)	
Diuretics	28 (12.5%)	67 (21.4%)	0.008
Anticoagulants	6 (2.7%)	22 (7.1%)	0.025
Antacids	47 (20.9%)	96 (30.8%)	0.011
Hypolipidemic agents	68 (30.4%)	126 (40.4%)	0.017
β-blockers	44 (19.8%)	77 (24.7%)	0.186
Calcium antagonists	9 (4.0%)	44 (14.2%)	<0.001
ACEIs	14 (18.9%)	29 (22.5%)	0.050
Insulin	10 (4.5%)	29 (9.3%)	0.034
Oral antidiabetic agents	37 (16.5%)	61 (19.6%)	0.361
Bronchodilators	16 (7.1%)	12 (3.8%)	0.091

Values represent the percentage of the patients and control subjects or means ± standard deviations. AHI, apnea-hypopnea index; ODI, oxygen desaturation index; BMI, body mass index; HDL, high-density lipoprotein; LDL, low-density lipoprotein; ACEIs, angiotensin-converting enzyme inhibitors.

OSA was detected in 58% of the study subjects (312 patients and 226 controls).

Patients with OSA were significantly older and had higher BMIs compared with those without OSA. No difference was detected between genders (Table 1). The number of hypertensive patients was significantly higher in the OSA group (Table 1). The number of patients who took antihypertensive drugs, particularly diuretics and calcium agonists, was higher in the OSA group.

PlGF levels were higher in patients with OSA compared with patients without OSA (19.9 pg/mL, interquartile range: 16.6–24.5 pg/mL; 18.5 pg/mL, interquartile range: 14.7–22.7 pg/mL; p<0.001).

**Fig 1:**
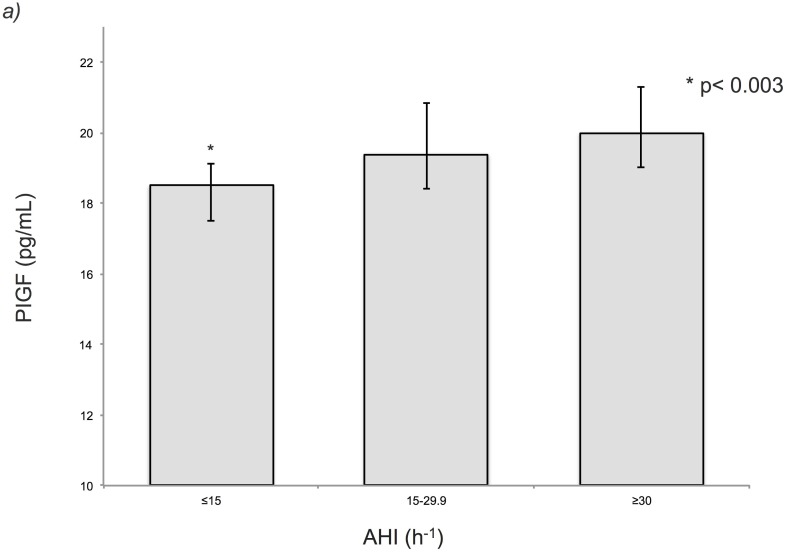
Levels of PlGF according to AHI groups. Error bars represent 95% confidence intervals. The Kruskal-Wallis test was used for comparisons among groups.

PlGF levels were correlated with age (r = 0.304, p<0.001), AHI (r = 0.142, p = 0.001), Min SaO_2_ (r = -0.091, p = 0.038), ODI (r = 0.128, p = 0.004), troponin concentration (r = 0.171, p<0.001) and mean peak troponin level (r = 0.161, p = 0.001). To investigate the strength of the association between PlGF levels and OSA severity, patients were classified into 3 groups according to AHI. The results showed that PlGF levels increased with AHI even after adjustment. PlGF levels were statistically different among the three groups and higher in the AHI≥30 group (19.9 pg/mL, interquartile range: 17.5–24.8) than both the AHI 15–29.9 group (19.3 pg/mL, interquartile range: 16.2–23.5) and the AHI≤15 group (18.2 pg/mL, interquartile range: 14.7–22.1); (p<0.003; Fig 1).

To further assess the associations of variables related to sleep and to the severity of ACS with PlGF levels, we classified the study participants into two groups according to PlGF concentrations (Table 2). A cutoff value of 20 pg/mL for PlGF levels was used based on prior studies [20]. Using this cut-off value, we found that up to 156 patients with OSA had PlGF levels >20, which represent 63.7% of patients with PlGF ≥20 pg/mL. By contrast, the number of non-OSA patients with PlGF levels ≥20 pg/mL was 81, which represent 36.3% of patients with PlGF ≥20 pg/mL.

**Table 2:** Baseline patient characteristics and variables related to the severity of acute coronary syndrome according to PlGF status.

	PlGF<20 pg/mL(n = 301)	PlGF≥20 pg/mL(n = 237)	p-value
Age, years	57.0 ± 11.0	61.8 ± 10.5	<0.001
Male, %	245(81.9%)	192 (81.6%)	0.927
OSA	160 (53.3%)	156 (63.7%)	0.015
BMI, kg•m^-2^	27.3 ± 6.3	27.5 ± 6.2	0.653
Hypertension	135 (44.7%)	132 (55.2%)	0.015
Diabetes mellitus	71 (23.6%)	61 (25.7%)	0.565
Dyslipidemia	167 (55.5%)	129 (54.4%)	0.808
Smoker			0.138
No	86 (28.5%)	75 (31.5%)	
Yes	147 (48.7%)	96 (40.3%)	
Former smoker	69 (22.8%)	67 (28.2%)	
Number of diseased vessels ≥3	48 (17.6%)	65 (30.2%)	<0.001
Killip score >1	12 (5.0%)	23 (12.9%)	0.004
Ejection fraction <51.5%*	58 (25.8%)	40 (23.4%)	0.586
Peak troponin level≥724.5 ng/mL*	57 (24.4%)	45 (26.0%)	0.704
CV complications during hospitalization	16 (5.15%)	23 (9.2%)	0.059

PlGF, placental growth factor; OSA, obstructive sleep apnea; BMI, body mass index; CV, cardiovascular.

*Optimal threshold to discriminate between OSA and control patients [16].

There were no differences in the trend for the ACS category and OSA (unstable angina was present in 14.8% of controls and 13.3% of OSA; NSTEMI in 42.6% of controls and 41.5% of OSA; STEMI in 42.6% of controls and 45.2% of OSA). No significant association was detected between ACS category and PlGF levels.

Patients with elevated PlGF levels were more frequently older and hypertensive, and with respect to ACS severity, the percentages of patients with Killip score >1 and with 3 or more diseased vessels were higher in the high-PlGF group (Table 2).

In a multivariable analysis, PlGF, together with all baseline characteristics that were found to be predictive in univariate analyses, persisted as independent predictors of severity and short-term outcome (Tables 3 and 4). Patients with higher levels of PlGF had increased odds ratios for a Killip classification >1 (Table 3, ORs 3.13, p = 0.009; and ORs 2.67, p = 0.011) and for the presence of 3 or more diseased vessels (Table 4, ORs 2.02, p = 0.001; and ORs 1.85, p = 0.009) even after adjustment.

**Table 3:** Multivariate adjusted odds ratios for Killip score >1.

	OR (95% CI) Not adjusted	p-value	OR (95% CI) Multivariate model^1^	p-value	OR (95% CI) Multivariate model^2^	p-value
Age	1.04 (1.01–1.08)	0.013	1.01 (0.97–1.05)	0.576	1.01 (0.97–1.05)	0.593
Gender	0.53 (0.24–1.15)	0.107	0.63 (0.26–1.55)	0.314	0.63 (0.26–1.54)	0.309
Smoking	0.54 (0.26–1.09)	0.087	0.85 (0.36–1.99)	0.702	0.89 (0.38–2.09)	0.781
BMI	1.00 (0.95–1.06)	0.906	0.98 (0.93–1.04)	0.547	0.98 (0.93–1.03)	0.456
Hypertension	3.82 (1.69–8.62)	0.001	2.59 (1.02–6.56)	0.045	2.32 (0.90–5.97)	0.080
Diabetes	2.47 (1.22–5.03)	0.012	1.79 (0.82–3.89)	0.141	1.80 (0.82–3.92)	0.143
Dyslipidemia	1.43 (0.70–2.88)	0.325	1.07 (0.51–2.27)	0.858	1.11 (0.52–2.37)	0.787
OSA	2.07 (0.97–4.41)	0.060	-	-	1.83 (0.80–4.20)	0.155
PlGF ≥20 pg/mL	3.13 (1.34–7.34)	0.009	2.67 (1.25–5.69)	0,011	2.70 (1.26–5.77)	0.011

^1^ Multivariate model adjusted for age, gender (male vs female), smoking (current or former smoker vs non-smoker), BMI, hypertension, diabetes, dyslipidemia.

^2^ Multivariate model adjusted for age, gender (male vs female), smoking (current or former smoker vs non-smoker), BMI, hypertension, diabetes, dyslipidemia and OSA.

**Table 4:** Multivariate adjusted odds ratios for ≥3 diseased vessels.

	OR (95% CI) Not adjusted	p-value	OR (95% CI) Multivariate model^1^	p-value	OR (95% CI) Multivariate model^2^	p-value
Age	1.03 (1.01–1.05)	0.005	1.02 (0.99–1.04)	0.213	1.01 (0.99–1,04)	0.264
Gender	1.94 (1.01–3.72)	0.047	2.84 (1.38–5.83)	0.005	2.86 (1.39–5.90)	0.004
Smoking	0.66 (0.42–1.04)	0.073	0.70 (0.42–1.16)	0.168	0.70 (0.42–1.17)	0.172
BMI	1.02 (0.98–1.06)	0.390	1.02 (0.98–1.06)	0.461	1.01 (0.97–1.06)	0.541
Hypertension	2.27 (1.47–3.50)	<0.001	1.60 (0.96–2.67)	0.070	1.56 (0.93–2.62)	0.088
Diabetes	2.78 (1.77–4.38)	<0.001	2.27 (1.38–3.75)	0.001	2.27 (1.37–3.74)	0.001
Dyslipidemia	1.61 (1.05–2.48)	0.030	1.34 (0.84–2.15)	0.219	1.35 (0.84–2.16)	0.213
OSA	1.57 (1.01–2.40)	0.045	-	-	1.25 (0.77–2.03)	0.367
PlGF level ≥20 ng/L	2.02 (1.32–3.10)	0.001	1.85 (1.16–2.94)	0.009	1.83 (1.15–2.91)	0.011

^1^ Multivariate model adjusted for age, gender (male vs female), smoking (current or former smoker vs non-smoker), BMI, hypertension, diabetes, dyslipidemia.

^2^ Multivariate model adjusted for age, gender (male vs female), smoking (current or former smoker vs non-smoker), BMI, hypertension, diabetes, dyslipidemia and OSA.

The number of cardiovascular complications (heart failure, reinfarction, mechanic complications, arrhythmia and stroke) tended to be higher in the high-PlGF group. However, the multivariate analysis revealed that despite the fact that patients with OSA had high PlGF levels, when assessing the associations between these ACS severity-related variables and high PlGF levels, there were no statistically significant differences after adjustment for OSA (Table 3 and Table 4). In addition, no significant interactions were seen between PlGF levels and the effect of OSA with respect to other clinical outcomes such as the length of stay in the coronary unit, length of hospitalization, ejection fraction, number of stents implanted or peak of troponin (p>0.05).

## Discussion

The results of this study show that in patients with ACS, elevated plasma levels of PlGF are associated with the presence of OSA and with adverse outcomes during short-term follow-up. The present findings suggest that the presence of OSA may affect the clinical significance of PlGF in patients with ACS.

PlGF is an established prognostic marker in ACS. However, no study has specifically examined its prognostic capabilities in OSA patients with ACS.

In a previous study, we observed that OSA influences the severity of ACS and its short-term prognosis. OSA was correlated with an increase in peak plasma troponin levels, with the number of diseased vessels and with the length of stay in the coronary unit [17]. In this study, we observed that in patients with ACS, higher PlGF levels were associated with adverse short-term outcomes (greater numbers of diseased vessels, higher Killip scores and a tendency to develop more cardiovascular complications). These observations suggest that different pathophysiological mechanisms may be responsible for the expression of PlGF in patients with and without OSA and that these mechanisms may affect short- and long-term risks after ACS in different ways.

PlGF, a member of the VEGF family of angiogenic proteins, plays an important role in pathological angiogenesis [21]. Although PlGF and VEGF activate similar signaling pathways, PlGF exhibits greater disease-specific activity than VEGF does, while it does not affect quiescent vessels in healthy tissues. In addition to enhancing angiogenesis, PlGF is known to improve cardiac performance by promoting cardiomyocyte survival and cardiomyogenesis via recruitment of bone marrow-derived progenitor cells towards infarcted myocardial tissue [22]. Hypoxia is an important stimulus of PlGF expression, and hypoxia inducible factor-1-alpha (HIF-1α) can directly activate its transcription [10;13]. High PlGF release typically accompanies acute ischemia and infarction, but elevated levels may reflect underlying acute or chronic hypoxia. Our results demonstrate that OSA is an important determinant of PlGF levels in patients with ACS. Among several confounding factors, OSA seems to mediate a portion of the release of PlGF detected in these patients. In line with this data, PlGF may represent a valuable marker of OSA in patients suspected of having acute coronary syndrome. Furthermore, the presence of OSA may affect the prognostic value of PlGF levels, and this possibility should be considered in studies in which PlGF is used as a clinical biomarker for risk stratification.

There is evidence that OSA may be associated with the activation of cardiovascular adaptive mechanisms. Berger et al reported that the numbers of endothelial progenitor cells are elevated and that angiogenesis increases in patients with AMI and coexistent OSA compared with patients with AMI without OSA [6]. EPCs are mobilized by signaling pathways, such as the HIF-1α pathway, which are also activated in OSA [23] [24]. HIF-1α stimulates the production of VEGF, and several studies have shown that patients with OSA have increased levels of VEGF [25]. In contrast, another study found that although VEGF expression in monocytes was found to be higher in patients with AMI and OSA compared with patients with AMI without OSA, no difference was reported for plasma VEGF levels between these groups [6]. On the other hand, earlier studies, which demonstrated that PlGF levels at presentation are of prognostic value for clinical outcomes in patients with ACS, did not find any correlation between PlGF levels and VEGF levels [12]. Thus far, the effect of OSA on plasma PlGF levels after ACS has not been investigated. In patients with ACS, plasma levels of PlGF increase acutely and transiently [12]. A single initial measurement of a patient’s plasma PlGF level appears to extend the predictive and prognostic information gained from traditional risk markers [26]. The extent to which PlGF levels are elevated is influenced by the severity of myocardial damage, and the overall effects of PlGF may vary with disease status and comorbidities [27–29]. In this sense, OSA could be essential and may mediate a portion of the prognostic impact of PlGF, given the relationship between PlGF levels after ACS and OSA that was observed in our study. However, it is known that OSA increases the incidence of morning peak of onset in acute myocardial infarction. Despite the fact that there are no data on whether PlGF has a diurnal pattern of variation, a perturbation in circadian PlGF balance might be a possible contributor to the onset of MI [30;31]. Furthermore, elevated PlGF levels may reflect underlying acute or chronic hypoxia. PlGF has potent angiogenic properties, especially under pathological circumstances, and increased PlGF levels could counteract the damaging effects of ischemia [21]. Iwama et al observed that patients with higher plasma PlGF levels on day 3 after AMI showed greater improvement in left ventricular ejection fraction (LEVF) during the chronic phase (6 months post MI) than did patients with lower plasma PlGF levels, and they also observed that patients with improvement in LEVF in the chronic phase had significantly higher plasma levels of PlGF in the acute phase compared with patients without improvement; these findings suggest that PlGF may be involved in repairing injured myocardial tissue [13]. In addition, experimental studies have demonstrated that PlGF may serve both as a marker of adaptive cardiac remodeling and as a promising novel therapeutic agent for revascularizing and regenerating the infarcted myocardium and for improving its performance after MI [14;22]. Consistent with these findings, it is possible that the elevated levels of PlGF detected in the group of patients with OSA could exert a beneficial effect that could promote long-term improvement in cardiac function after ACS in these patients.

### Limitations

This study has several limitations. First, we excluded patients with daytime sleepiness, which were the patients who exhibited the most severe OSA. Second, OSA was diagnosed based on respiratory polygraphy, which could underestimate the severity of OSA. Third, the high variability in PlGF levels detected both in controls and patients may limit its usefulness. On the other hand, this study was performed only for patients with ACS. Previous clinical studies including subjects without ACS showed PlGF mean plasma levels of 10 pg/mL [13] and 16.6 pg/mL [31], both of them lower than in our ACS patients groups, either with or without OSA. Ideally, longitudinal studies measuring PlGF at the time of diagnosis of OSA would add to the verification of PlGF as a biomarker. Fourth, alternative noninvasive biomarkers of cardiac dysfunction, such as the brain natriuretic peptide (BNP), were not assessed. As reported elsewhere, PlGF and BNP levels are known to positively correlate in patients with ischemic cardiomyopathy [32]. This demonstrates an increase of PlGF with the severity of heart failure in such patients, regardless of OSA. Finally, the design of this study does not allow us to evaluate the long-term prognostic role of PlGF in OSA or to draw definitive conclusions.

## Conclusions

The results of this study show that in patients with ACS, elevated plasma levels of PlGF are associated with the presence of OSA and with adverse outcomes during short-term follow-up. These findings suggest that different pathophysiological mechanisms might affect the cardiac expression of PlGF after ischemic injury, as well as their predictive role in patients with and without OSA.

## Abbreviations

ACS: Acute coronary syndrome
AHI: Apnea-hypopnea index
CPAP: Continuous positive airway pressure
AMI: Acute myocardial infarction
ESS: Epworth sleepiness scale
BMI: Body mass index
CU: Coronary unit
CV: Cardiovascular
ODI: Oxygen desaturation index
OSA: Obstructive sleep apnea
MI: Myocardial infarction
PlGF: Placental growth factor
SaO2: Oxygen saturation
OR: odds ratio
STEMI: ST segment elevation myocardial infarction
VEGF: Vascular endothelial growth factor

## References

[1] Ludka O, Stepanova R, Vyskocilova M, Galkova L, Mikolaskova M, et al: Sleep apnea prevalence in acute myocardial infarction—the Sleep Apnea in Post-acute Myocardial Infarction Patients (SAPAMI) Study. Int J Cardiol 2014;176:13–19. 10.1016/j.ijcard.2014.06.020 25064202 PMC4249636

[2] Sanchez-de-la-Torre M, Campos-Rodriguez F, Barbe F: Obstructive sleep apnoea and cardiovascular disease. Lancet Respir Med 2013;1:61–72. 10.1016/S2213-2600(12)70051-6 24321805

[3] Kohler M, Stradling JR: Mechanisms of vascular damage in obstructive sleep apnea. Nat Rev Cardiol 2010;7:677–685. 10.1038/nrcardio.2010.145 21079639

[4] Kuniyoshi FH, Garcia-Touchard A, Gami AS, Romero-Corral A, van der WC, Pusalavidyasagar S, et al: Day-night variation of acute myocardial infarction in obstructive sleep apnea. J Am Coll Cardiol 2008;52:343–346. 10.1016/j.jacc.2008.04.027 18652941 PMC2598735

[5] Shah N, Redline S, Yaggi HK, Wu R, Zhao CG, Ostfeld R, et al: Obstructive sleep apnea and acute myocardial infarction severity: ischemic preconditioning? Sleep Breath 2012. 10.1007/s11325-012-0770-7 23090861

[6] Berger S, Aronson D, Lavie P, Lavie L: Endothelial progenitor cells in acute myocardial infarction and sleep-disordered breathing. Am J Respir Crit Care Med 2013;187:90–98. 10.1164/rccm.201206-1144OC 23155141

[7] Ramasamy I: Biochemical markers in acute coronary syndrome. Clin Chim Acta 2011;412:1279–1296. 10.1016/j.cca.2011.04.003 21501603

[8] Kehl DW, Iqbal N, Fard A, Kipper BA, De La Parra LA, Maisel AS: Biomarkers in acute myocardial injury. Transl Res 2012;159:252–264. 10.1016/j.trsl.2011.11.002 22424429

[9] Carmeliet P, Moons L, Luttun A, Vincenti V, Compernolle V, De MM, et al: Synergism between vascular endothelial growth factor and placental growth factor contributes to angiogenesis and plasma extravasation in pathological conditions. Nat Med 2001;7:575–583. 11329059 10.1038/87904

[10] Liu X, Claus P, Wu M, Reyns G, Verhamme P, Pokreisz P, et al: Placental growth factor increases regional myocardial blood flow and contractile function in chronic myocardial ischemia. Am J Physiol Heart Circ Physiol 2013;304:H885–H894. 10.1152/ajpheart.00587.2012 23316060

[11] Carnevale D, Lembo G: Placental growth factor and cardiac inflammation. Trends Cardiovasc Med 2012;22:209–212. 10.1016/j.tcm.2012.07.022 22925712

[12] Heeschen C, Dimmeler S, Fichtlscherer S, Hamm CW, Berger J, Simoons ML, et al: Prognostic value of placental growth factor in patients with acute chest pain. JAMA 2004;291:435–441. 14747500 10.1001/jama.291.4.435

[13] Iwama H, Uemura S, Naya N, Imagawa K, Takemoto Y, Asai O, et al: Cardiac expression of placental growth factor predicts the improvement of chronic phase left ventricular function in patients with acute myocardial infarction. J Am Coll Cardiol 2006;47:1559–1567. 16630991 10.1016/j.jacc.2005.11.064

[14] Accornero F, van Berlo JH, Benard MJ, Lorenz JN, Carmeliet P, Molkentin JD: Placental growth factor regulates cardiac adaptation and hypertrophy through a paracrine mechanism. Circ Res 2011;109:272–280. 10.1161/CIRCRESAHA.111.240820 21636802 PMC3146170

[15] Esquinas C, Sanchez-de-la Torre M, Aldoma A, Flores M, Martinez M, Barcelo A, et al: Rationale and methodology of the impact of continuous positive airway pressure on patients with ACS and nonsleepy OSA: the ISAACC Trial. Clin Cardiol 2013;36:495–501. 10.1002/clc.22166 23843147 PMC6649460

[16] Berry RB, Budhiraja R, Gottlieb DJ, Gozal D, Iber C, Kapur VK, et al: Rules for scoring respiratory events in sleep: update of the 2007 AASM Manual for the Scoring of Sleep and Associated Events. Deliberations of the Sleep Apnea Definitions Task Force of the American Academy of Sleep Medicine. J Clin Sleep Med 2012;8:597–619. 10.5664/jcsm.2172 23066376 PMC3459210

[17] Barbe F, Sanchez-de-la-Torre A, Abad J, Duran-Cantolla J, Mediano O, Amilibia J, et al: Effect of obstructive sleep apnoea on severity and short-term prognosis of acute coronary syndrome. Eur Respir J 2015. 10.1183/09031936.00071714 25573410

[18] Spanish national Consensus in sleep Apnea-hypopnea syndrome(SAHS): Arch Bronconeumol 2005;41:7–9. 10.1016/s1579-2129(06)60248-6 16029734

[19] Chiner E, Arriero JM, Signes-Costa J, Marco J, Fuentes I: [Validation of the Spanish version of the Epworth Sleepiness Scale in patients with a sleep apnea syndrome]. Arch Bronconeumol 1999;35:422–427. 10596338 10.1016/s0300-2896(15)30037-5

[20] Hochholzer W, Reichlin T, Stelzig C, Hochholzer K, Meissner J, Breidthardt T, et al: Impact of soluble fms-like tyrosine kinase-1 and placental growth factor serum levels for risk stratification and early diagnosis in patients with suspected acute myocardial infarction. Eur Heart J 2011;32:326–335. 10.1093/eurheartj/ehq429 21138939

[21] Accornero F, Molkentin JD: Placental growth factor as a protective paracrine effector in the heart. Trends Cardiovasc Med 2011;21:220–224. 10.1016/j.tcm.2012.05.014 22902069 PMC3424519

[22] Iwasaki H, Kawamoto A, Tjwa M, Horii M, Hayashi S, Oyamada A, et al: PlGF repairs myocardial ischemia through mechanisms of angiogenesis, cardioprotection and recruitment of myo-angiogenic competent marrow progenitors. PLoS One 2011;6:e24872 10.1371/journal.pone.0024872 21969865 PMC3182165

[23] Arnardottir ES, Mackiewicz M, Gislason T, Teff KL, Pack AI: Molecular signatures of obstructive sleep apnea in adults: a review and perspective. Sleep 2009;32:447–470. 19413140 10.1093/sleep/32.4.447 PMC2663860

[24] de la Peña M, Barcelo A, Barbe F, Pierola J, Pons J, Rimbau E, et al: Endothelial function and circulating endothelial progenitor cells in patients with sleep apnea syndrome. Respiration 2008;76:28–32. 17921670 10.1159/000109643

[25] Gozal D, Lipton AJ, Jones KL: Circulating vascular endothelial growth factor levels in patients with obstructive sleep apnea. Sleep 2002;25:59–65. 11833862 10.1093/sleep/25.1.59

[26] Bui AH, Bonaca MP, Sabatine MS, Ray KK, Rifai N, Cannon CP, et al: Elevated concentration of placental growth factor (PlGF) and long term risk in patients with acute coronary syndrome in the PROVE IT-TIMI 22 trial. J Thromb Thrombolysis 2012;34:222–228. 10.1007/s11239-012-0704-z 22446996

[27] Tarnow L, Astrup AS, Parving HH: Elevated placental growth factor (PlGF) predicts cardiovascular morbidity and mortality in type 1 diabetic patients with diabetic nephropathy. Scand J Clin Lab Invest Suppl 2005;240:73–79. 16112962 10.1080/00365510500235970

[28] Theilade S, Lajer M, Jorsal A, Tarnow L, Parving HH, Rossing P: Evaluation of placental growth factor and soluble Fms-like tyrosine kinase 1 as predictors of all-cause and cardiovascular mortality in patients with Type 1 diabetes with and without diabetic nephropathy. Diabet Med 2012;29:337–344. 10.1111/j.1464-5491.2011.03482.x 21988672

[29] Lenderink T, Heeschen C, Fichtlscherer S, Dimmeler S, Hamm CW, et al: Elevated placental growth factor levels are associated with adverse outcomes at four-year follow-up in patients with acute coronary syndromes. J Am Coll Cardiol 2006;47:307–311. 16412852 10.1016/j.jacc.2005.08.063

[30] Nakashima H, Henmi T, Minami K, Uchida Y, Shiraishi Y, Nunohiro T, et al: Obstructive sleep apnoea increases the incidence of morning peak of onset in acute myocardial infarction. Eur Heart J Acute Cardiovasc Care 2013;2:153–158. 10.1177/2048872613478557 24222825 PMC3821804

[31] Bagai K, Muldowney JA III, Song Y, Wang L, Bagai J, Artibee KJ, et al: Circadian variability of fibrinolytic markers and endothelial function in patients with obstructive sleep apnea. Sleep 2014;37:359–367. 10.5665/sleep.3414 24497664 PMC3900618

[32] Nakamura T, Funayama H, Kubo N, Yasu T, Kawakami M, Momomura S, et al: Elevation of plasma placental growth factor in the patients with ischemic cardiomyopathy. Int J Cardiol 2009;131:186–191. 10.1016/j.ijcard.2007.10.050 18192038

